# LiSA-MobileNetV2: an extremely lightweight deep learning model with Swish activation and attention mechanism for accurate rice disease classification

**DOI:** 10.3389/fpls.2025.1619365

**Published:** 2025-08-08

**Authors:** Yongqi Xu, Dongcheng Li, Changcheng Li, Zheming Yuan, Zhijun Dai

**Affiliations:** Hunan Engineering and Technology Research Center for Agricultural Big Data Analysis & Decision-making, Hunan Agricultural University, Changsha, China

**Keywords:** disease recognition, rice, lightweight CNN, attention mechanism, activation function

## Abstract

In the context of intelligent agriculture in China, rapid and accurate identification of crop diseases is essential for ensuring food security and improving crop yield. Although lightweight convolutional neural networks (CNNs) are widely adopted for plant disease recognition due to their computational efficiency, they often suffer from limited feature representation and classification accuracy. To address these challenges, we propose LiSA-MobileNetV2, an improved MobileNetV2-based model designed for rice disease classification. First, we restructure the inverted residual module to simplify the network architecture, achieving a test accuracy of 92.32%, representing a 2.41% improvement over the original MovileNetV2 (89.91%). This indicate that a more lightweight network can enhance feature representation in specific disease recognition. Second, integrating the Swish activation function further improves accuracy to 94.04% by enhancing the model’s non-linear feature learning. Finally, the addition of a squeeze-and-excitation attention mechanism raises accuracy to 95.68%, representing a 5.77% improvement over the original model. Importantly, the parameter size and FLOPs are reduced by 74.69% and 48.18%, respectively, maintaining strong computational efficiency. These results demonstrate that combining structural simplification, advanced activation, and efficient attention mechanisms significantly improves CNN performance. LiSA-MobileNetV2 provides a high-accuracy, resource-efficient solution for real-time rice disease detection in smart farming systems.

## 1 Introduction

Rice is one of the most important staple crops globally, especially in Asian, where it serves as the primary food source for hundreds of millions of people (Xu et al., 2021). However, diseases such as rice blast, bacterial leaf blight, sheath blight, and brown spot seriously threat both yield and quality during rice cultivation (Xiao et al., 2021; Rezvi et al., 2023). These diseases not only reduce production but can also cause large-scale economic losses, undermining food security and sustainable agricultural development (Deng et al., 2023). Therefore, rapid and accurate detection of rice diseases is crucial for effective prevention and control, safeguarding food security, and promoting agricultural modernization.

Traditional methods for detecting rice diseases and pests mainly rely on manual visual inspection, which is time-consuming, labor-intensive, and prone to misjudgment or omissions due to observer inexperience (Sankaran et al., 2010). In addition, manual detection has low efficiency, making it inadequate for the large-scale, high-throughput needs of modern agriculture (Xu et al., 2024). With advancements in computer technology, traditional image processing and machine learning methods have been applied to agricultural disease detection (Kaundal et al., 2006). These methods relying on image preprocessing, feature extraction, and classification algorithms have improved detection efficiency to some extent (Mukherjee et al., 2025). However, their reliance on manually designed features and their limited generalization ability constrain practical applications (Deng et al., 2021). For instance, traditional machine learning often requires complex, disease-specific preprocessing and segmentation and struggles to capture underlying image patterns, limiting its scalability in agricultural production (Sanyal and Patel, 2008).

In recent years, the rapid development of artificial intelligence and computer vision technologies has promoted the application of deep learning in crop disease and pest identification. Compared with traditional methods, deep learning models, especially convolutional neural networks (CNNs), can automatically learn high-level semantic features from large-scale image data, effectively improving both accuracy and efficiency in rice disease and pest recognition (Khan et al., 2020; Li et al., 2024). CNNs offer powerful feature representation and generalization ability, enabling them to handle complex agricultural scenarios and accurately identify disease patterns (Salman et al., 2023). To further improve model performance in challenging environments, attention mechanisms have been introduced. These mechanisms help models focus on disease-related regions, thereby boosting classification accuracy (Niu et al., 2021). For instance, spatial attention emphasizes key regions in an image to improve localization and recognition (Zhu et al., 2019), while channel attention dynamically adjusts the importance of different feature channels to better represent disease characteristics (Karthik et al., 2022).

Recent advancements in intelligent agriculture have demonstrated the potential of integrating sensing, modeling, and AI-driven analysis for improving crop monitoring and management. For instance, a 3D point cloud-based phenotyping method enabled non-destructive and high-throughput trait extraction in Chinese Cymbidium seedlings (Zhou et al., 2024), highlighting the importance of automated data acquisition in precision agriculture, though it focused on morphology rather than pathology. Similarly, pair‐wise comparison analysis for multiple pool‐seq (PCAMP) was used to identify anthocyanin biosynthesis genes in rice pericarp, uncovering genetic factors responsible for color variation (Yang et al., 2019); while our work does not address genomic data, such work highlights the potential of integrating genetic and visual information for comprehensive crop health monitoring. Beyond plant traits, advances in agrochemical design, such as the use of cyclopropane with triangular stable structures to enhance pesticide stability, offer a complementary perspective on crop protection strategies (Huang et al., 2025). Deep learning has also been widely adopted in agricultural information systems; for example, deep belief networks have supported IoT-based frameworks for predicting vegetable market trends (Luo et al., 2022), underscoring the versatility of AI technologies across agricultural domains.

Although deep learning has made remarkable progress in rice disease detection, significant challenges remain in practical applications. The robustness of lightweight models, such as the MobileNet series, still requires improvement in complex field environments (Zhang et al., 2023). Identification deviations may arise due to overlapping symptoms, lighting variations, and background interference (Yang et al., 2023). Furthermore, limited computing resources and inference efficiency constraints hinder model deployment in real-world agricultural production.

To address these challenges, we propose LiSA-MobileNetV2 (Lightweight MobileNetV2 with Swish activation and squeeze-and-excitation Attention module), a novel lightweight visual recognition framework for real-time rice disease detection. Unlike prior studies focusing on phenotypic or molecular traits, our approach combines an optimized deep convolutional architecture with attention mechanisms designed for field imagery, aiming to balance computational efficiency and high classification accuracy. Specifically, we restructure the inverted residual block of MobileNetV2, replace the ReLU6 activation with Swish to enhance nonlinear representation, and incorporate SE attention to better localize disease-relevant regions, thereby improving classification performance.

## 2 Materials and methods

### 2.1 Description of the rice disease dataset

**Figure 1:**
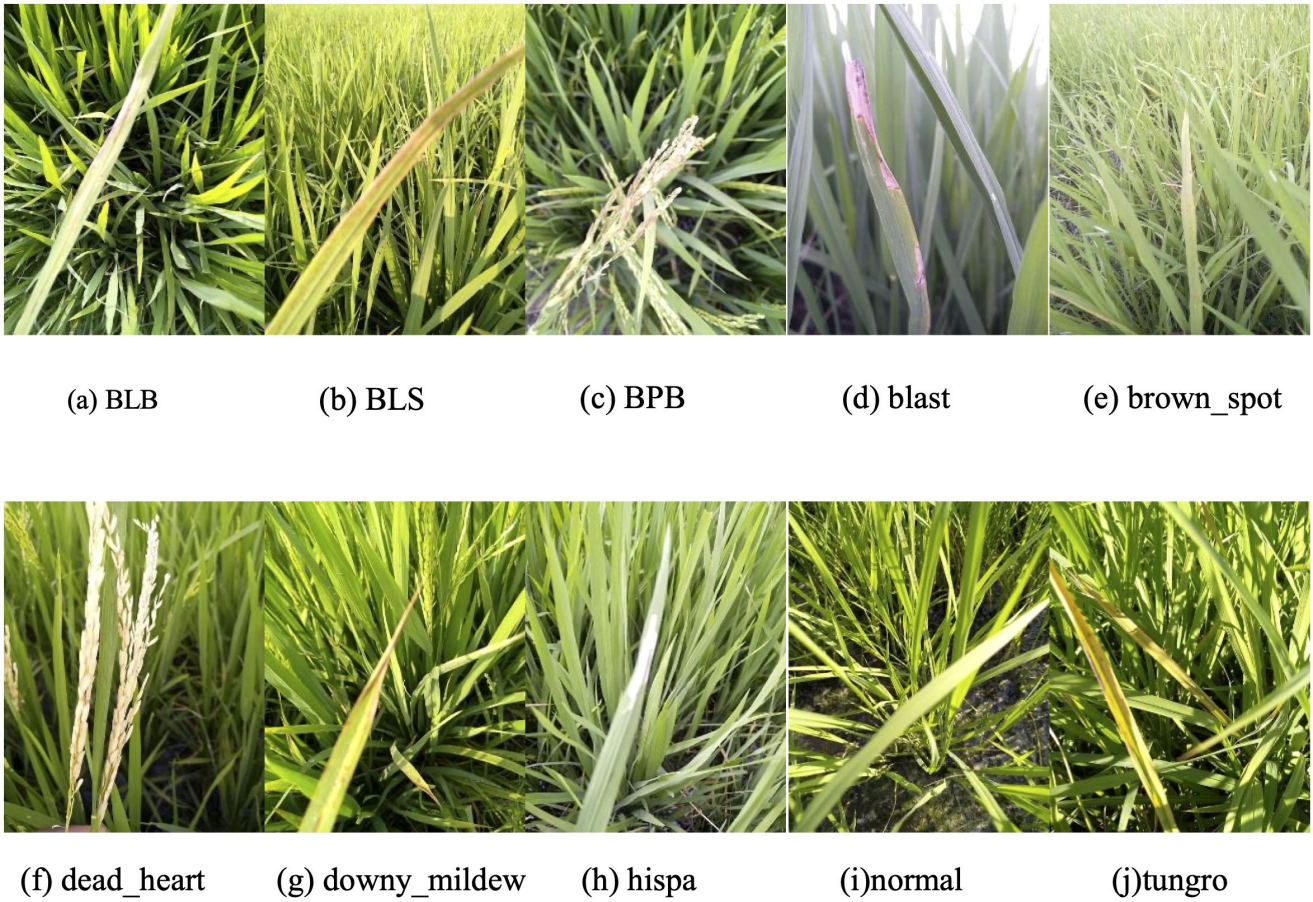
Examples images of the rice disease and healthy (normal) leaves utilized in this study. Categories were presented in alphabetical order. **(a)** BLB: bacterial leaf blight; **(b)** BLS: bacterial leaf streak; **(c)** BPB: bacterial panicle blight; **(d–h, j)** the other six diseases; **(i)** healthy leaf.

The dataset used in this study was derived from Paddy Doctor: A Visual Image Dataset for Automated Paddy Disease Classification and Benchmarking (Petchiammal et al., 2023). It comprises RGB color images (480×640 pixels) representing nine rice diseases, along with healthy rice samples (**Figure 1**). The ten classification categories include: bacterial leaf blight (BLB), bacterial leaf streak (BLS), bacterial panicle blight (BPB), blast, brown spot, dead heart, downy mildew, hispa, tungro, and normal (healthy). In total, the dataset contains 10,407 images. A stratified random sampling strategy was employed to partition the data: 10% was allocated as the test set for final performance evaluation, while the remaining 90% was further split into training (80%) and validation (20%) subsets. The training set was used for model fitting and parameter optimization, while the validation set guided hyperparameter tuning and monitored training progress. The distribution of samples across subsets was presented in **Table 1**.

**Table 1:** Sample size and oversampling coefficient for each rice disease class.

Class	Training set	Validation set	Test set	Oversampling coefficient ^a^
BLB	345	86	48	3.0
BLS	274	68	38	3.5
BPB	243	60	34	4.0
blast	1251	313	174	–
brown_spot	694	174	97	1.5
dead_heart	1038	260	144	–
downy_mildew	446	112	62	2.8
hispa	1148	287	159	–
normal	1270	318	176	–
tungro	783	196	109	1.4
Total	7492	1874	1041	

^a^The augmentation ratio indicating the oversampling factor applied to minority disease classes in the training set. BLB, bacterial leaf blight; BLS, bacterial leaf streak; BPB, bacterial panicle blight.

### 2.2 Data augmentation

As shown in **Table 1**, the dataset exhibits clear class imbalance. In particular, the training sample sizes for *blast*, *hispa*, and *normal* are substantially larger, with 1251, 1148, and 1270 images, respectively. Conversely, *BPB*, *BLS*, and *BLB* have the fewest samples, with only 243, 274, and 345 images, while *brown_spot* and *tungro* also contain relatively limited samples (694 and 783 images, respectively), all well below the average. Such imbalance adversely affects model training, as minority classes may lack sufficient representation for effective feature learning, leading to reduced classification performance. To mitigate this issue, an oversampling-based class balancing strategy was applied. Oversampling is a widely used approach for handling class imbalance, with common techniques including Random Oversampling (He and Garcia, 2009) and the Synthetic Minority Over-sampling Technique (SMOTE) (Efendiev et al., 2014). In this study, minority class samples were replicated based on predefined ratios to equalize the training data distribution (**Table 1**).

To ensure diversity among oversampled data, geometric spatial transformations including random rotation (± 10°), translation (± 10%), scaling (± 5%), and mirror filling were applied. These augmentations, generated in real time, guide the model to learn key features such as rotational and scale invariance, further alleviating class imbalance. Importantly, dataset splitting into training, validation, and test sets was performed before oversampling and augmentation, and these operations were applied exclusively to the training set to prevent data leakage.

### 2.3 Architecture of the MobileNetV2 baseline model

The baseline model in this study is based on MobileNetV2, a lightweight CNN designed for high computational efficiency and low resource consumption (Sandler et al., 2018). The core building block of MobileNetV2 is the Inverted Residual Block, which integrates three key components: an expansion convolution, a depthwise separable convolution, and a projection convolution (Zhu et al., 2024).

**Figure 2:**
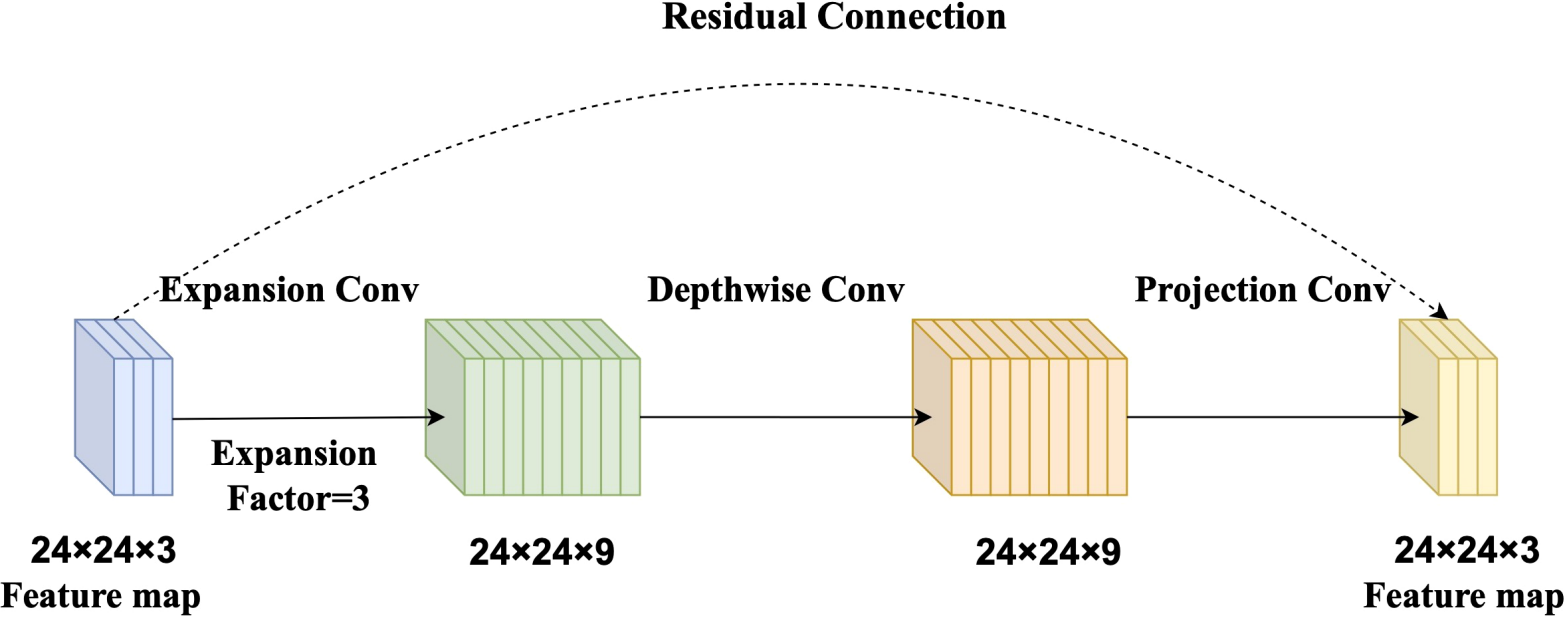
Diagram of the Inverted Residual Block. The expansion convolution (with an expansion factor of 3) increases channel dimensionality to enrich feature representation. The depthwise separable convolution extracts spatial features with reduced computational cost. The projection convolution reduces the number of channels, minimizing parameters and enhancing inference speed.

The expansion convolution first increases the number of channels to enrich feature representation. Subsequently, the depthwise separable convolution efficiently extracts spatial features while significantly reducing computational cost. Finally, the projection convolution compresses the feature map back to a lower-dimensional space, reducing the number of parameters and improving inference speed (**Figure 2**). This structure strikes a balance between model accuracy and computational efficiency, making it suitable for rice disease detection in resource-constrained agricultural settings.

### 2.4 Development of the LiSA-MobileNetV2 model

#### 2.4.1 Simplification of model architecture

**Figure 3:**
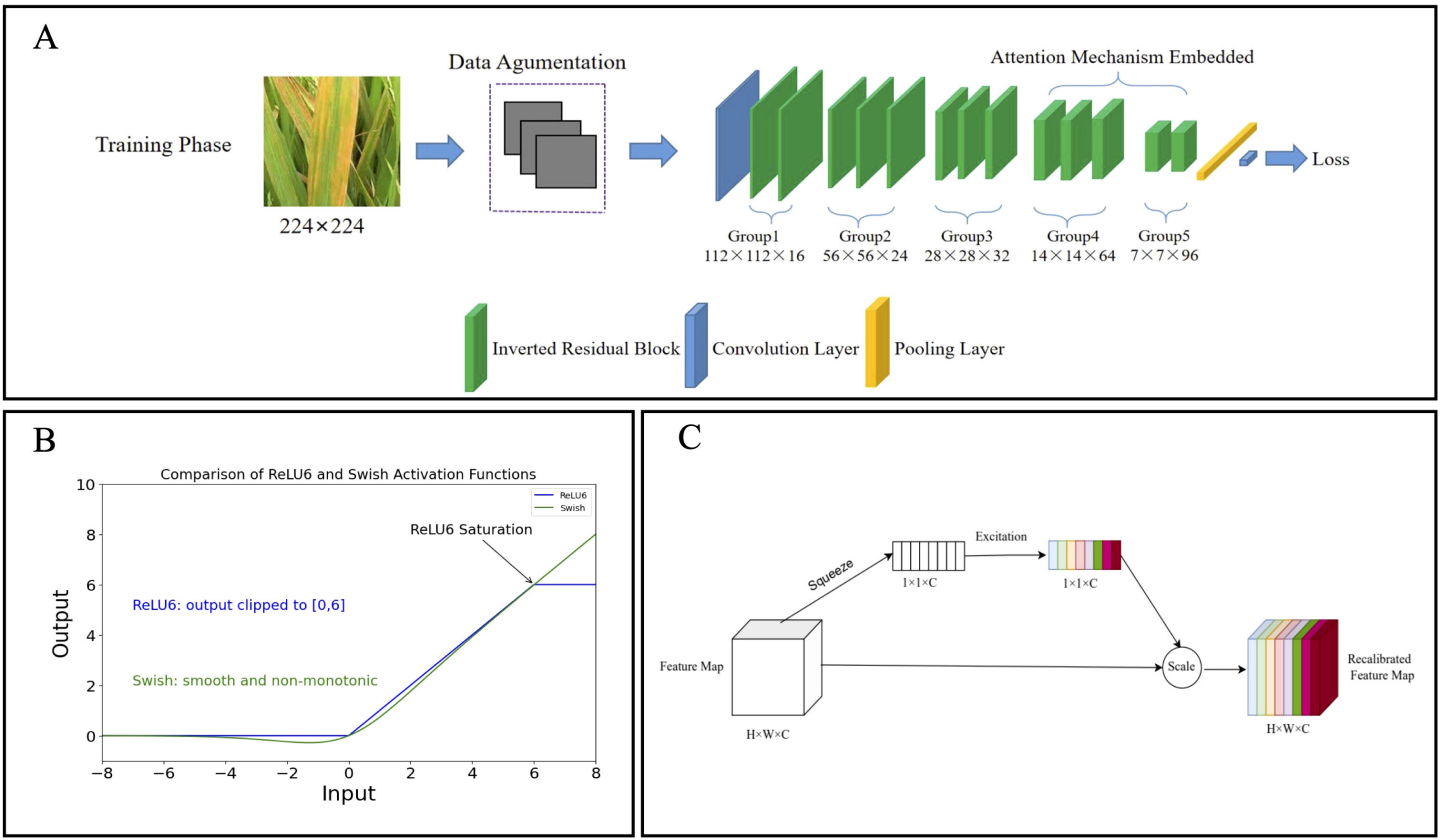
Diagram of the LiSA-MobilNetV2 network structure. **(A)** The inverted residual blocks (green) were reduced to 13 in total, forming five groups, with each group consisting of two or three blocks to create a more compact architecture. The SE attention modules were embedded in Group 4 and Group 5 to capture channel-wise feature dependencies, thereby improving the model’s ability to focus on disease-relevant regions. **(B)** Curves of the Swish and ReLU6 activation functions, where the Swish exhibits a smooth, non-monotonic transition without an upper bound, facilitating better gradient flow. **(C)** Schematic of the Squeeze-and-Excitation (SE) attention module. The module applies global average pooling for channel squeeze and a small fully connected network for channel excitation, producing recalibration weights for feature refinement.

In this study, the module configuration of MobileNetV2 was tailored to better meet the feature extraction requirements for rice disease imagery. The key modifications include: (1) adjusting the number of channels in each layer (ranges from 16×16 to 96×96) and modifying the expansion factor in the inverted residual blocks (from 2× to 8×, representing the ratio of output to input channels in the expansion layer); and (2) reducing the total number of inverted residual blocks from 19 to 13 to construct a more compact and efficient architecture (**Figure 3A**).

#### 2.4.2 Improvement of activation function

The activation function of the original MobileNetV2 was improved by replacing ReLU6 with Swish. ReLU6, a variant of ReLU, constrains its output within the range [0, 6] to limit activation magnitude and mitigate gradient explosion. However, this hard clipping may result in the loss of gradient information, thereby limiting the model’s learning capacity (Zhang et al., 2022). The Swish activation function is defined as follows:


(1) $$\begin{array}{c} Swish(x)=x\cdot \sigma (x) \end{array}$$

(2) $$\begin{array}{c} \sigma (x)=\frac{1}{1+e^{-x}} \end{array}$$

where σ(x) represents the sigmoid function, which maps the input to the range (0, 1).

Unlike ReLU6, Swish (Equations 1, 2) provides a smooth, non-monotonic without an upper bound constraint, allowing more effective gradient flow and better preservation of information during backpropagation (Zhang et al., 2022). This helps improve training stability, convergence speed, and generalization capability. Accordingly, Swish is employed in this study to replace ReLU6 (**Figure 3B**).

#### 2.4.3 Involvement of attention mechanism

Since standard inverted residual blocks may not adequately capture channel interdependencies, the Squeeze-and-Excitation (SE) attention module (Hu et al., 2018) was integrated into Group4 and Group5 of the inverted residual blocks in the network (**Figure 3A**). The core structure of the SE module is illustrated in **Figure 3C** and consists of two key operations (Equations 3, 4):

(1) Channel Squeeze: The three-dimensional feature map (H×W×C) is compressed into a one-dimensional channel descriptor (1×1×C) through global average pooling:


(3) $$\begin{array}{c} z_{c}=F_{sq}(u_{c})=\frac{1}{H\times W}\sum_{i=1}^{H}\sum_{j=1}^{W}u_{c}(i,j) \end{array}$$

where u_c_ (i,j) represents the activation at position (*i*, *j*) in the *c*-th channel.

(2) Channel Excitation: A small fully connected network is applied to the aggregated vector *z* to generate channel-wise importance weights:


(4) $$\begin{array}{c} s=F_{ex}(z)=\sigma (W_{2}\delta (W_{1}z)) \end{array}$$

where **W_1_** and **W_2_** are learnable parameters, *δ*(•) is the activation function, and *σ*(•) is the sigmoid function. The resulting weights ***s*** are used to recalibrate the original feature map through element-wise multiplication, thereby enhancing the network’s focus on informative features.

#### 2.4.4 Overview of model variants

To systematically evaluate the individual and combined contributions of architectural simplification, activation function enhancement, and attention mechanisms, four variants of MobileNetV2 were designed and compared in this study (**Table 2**):

1. Li-MobileNetV2 (Lightweight MobileNetV2): This variant employs a simplified MobileNetV2 structure with reduced initial convolutional channels and optimized inverted residual blocks, while retaining the original ReLU6 activation function.
2. LiS-MobileNetV2 (Lightweight MobileNetV2 with Swish activation): This model builds upon Li-MobileNetV2 by replacing ReLU6 with the Swish activation function, aiming to improve gradient flow, learning capability, and convergence speed.
3. LiA-MobileNetV2 (Lightweight MobileNetV2 with SE Attention): In this variant, the SE attention mechanism is incorporated into selected inverted residual blocks of Li-MobileNetV2 to enhance channel-wise feature recalibration, while ReLU6 is preserved as the activation function.
4. LiSA-MobileNetV2 (Lightweight MobileNetV2 with Swish activation and SE Attention): This model combines both the Swish activation and SE attention mechanisms on top of Li-MobileNetV2, integrating the benefits of improved nonlinearity and attention-guided feature refinement.

**Table 2:** Summary of the structural characteristics of model variants.

Model	Simplified architecture	ReLU6 activation	Swish activation	SE attention
Li-MobileNetV2	✓	✓		
LiS-MobileNetV2	✓		✓	
LiA-MobileNetV2	✓	✓		✓
LiSA-MobileNetV2	✓		✓	✓

### 2.5 Model evaluation and experimental setup

To assess the performance of the LiSA-MobileNetV2 model for rice disease recognition, several commonly used metrics were adopted, including Accuracy, Precision, FLOPs (floating-point operations), and Params (the total number of model parameters). Each metric provides a distinct perspective. Accuracy measures the proportion of correctly classified samples among all samples (Equation 5). Precision assesses the proportion of true positive predictions among all positive predictions made by the model (Equation 6). Recall measures the proportion of actual positive samples that are correctly identified by the model (Equation 7). F1-score is the harmonic mean of Precision and Recall, offering a balanced evaluation when both false positives and false negatives are critical (Equation 8). FLOPs quantify the computational cost of a single forward pass by counting the number of floating-point operations (Equation 9). Params reflects the total number of trainable parameters in the model, indicating its memory footprint and complexity. Additionally, confusion matrix was used to visualize classification performance across all classes, providing insight into model behavior at the class level.


(5) $$\begin{array}{c} Accuracy=\frac{\sum_{i=1}^{k}TP_{i}}{N} \end{array}$$

(6) $$\begin{array}{c} Precision=\frac{TP}{TP+FP} \end{array}$$

(7) $$\begin{array}{c} Recall=\frac{\text{TP}}{\text{TP}+\text{FN}} \end{array}$$

(8) $$\begin{array}{c} F1-score=\frac{2\times Precision\times Recall}{Precision+Recall} \end{array}$$

(9) $$\begin{array}{c} FLOPs=2\times C_{in}\times C_{out}\times K_{h}\times K_{w}\times H_{out}\times W_{out} \end{array}$$

Among these metrics, TP (True Positive): the number of positive samples correctly predicted; TN (True Negative): the number of negative samples correctly predicted; FP (False Positive): the number of negative samples incorrectly classified as positive; FN (False Negative): the number of positive samples incorrectly classified as negative. *C*_in_ and *C*_out_: the number of input and output channels, respectively. *K*_h_ and *K*_w_: the height and width of the convolution kernel; *H*_out_ and *W*_out_: the height and width of the output feature map.

All experiments were conducted on a system running Microsoft Windows 10, equipped with an Intel Core i5-12400F CPU and an NVIDIA GeForce RTX 2080 GPU (8 GB VRAM). The software environment included Python 3.11, TensorFlow 2.10.0, CUDA 10.8, and cuDNN 8.2.1.

The training was performed with an initial batch size is 32 and an adaptive learning rate strategy. The Adam optimizer was used with an initial learning rate is 10^-3^. To optimize learning efficiency and prevent overfitting, the ‘ReduceLROnPlateau’ callback was used to reduce the learning rate by a factor of 0.1 if the validation loss did not decrease for 5 consecutive epochs, with a minimum learning rate set at 10^−6^. The ‘EarlyStopping’ mechanism was applied with a patience of 10 epochs to halt training if no improvement in validation loss was observed. The ‘ModelCheckpoint’ was employed to save the model with the best validation performance. Dropout layers were incorporated into all model variants to enhance generalization and prevent overfitting. The code implementing the LiSA-MobileNetV2 model is written in Python and is available at https://github.com/ZhijunBioinf/LiSA-MobileNetV2.

## 3 Results

### 3.1 Classification results of the LiSA-MobileNetV2

To comprehensively evaluate the performance of our proposed LiSA-MobileNetV2 model in rice disease recognition, a detailed classification report was generated on the test set. Overall, the model exhibits stable and high classification performance across all categories, with F1-score consistently above 0.90, demonstrating both strong discriminative power and robust generalization ability (**Table 3**).

**Table 3:** Classification performance of the proposed LiSA-MobileNetV2 model for each rice disease on the test set.

Class	Precision	Recall	F1-score	Support
BLB	0.9375	0.9375	0.9375	48
BLS	1.0000	0.9737	0.9867	38
BPB	1.0000	0.9706	0.9851	34
blast	0.9282	0.9655	0.9465	174
brown_spot	0.9479	0.9381	0.9430	97
dead_heart	0.9931	0.9931	0.9931	144
downy_mildew	0.9636	0.8548	0.9060	62
hispa	0.9212	0.9560	0.9383	159
normal	0.9770	0.9659	0.9383	176
tungro	0.9630	0.9541	0.9585	109

Specifically, the model performs most excellently on “dead_heart”, achieving a precision, recall, and F1-score of 0.9931, indicating near-perfect classification. Similarly, “BLS” (bacterial_leaf_streak) and “BPB” (bacterial_panicle_blight) also exhibit excellent performance, with F1-score close to 0.99, reflecting the model’s exceptional capability in identifying minority diseases after augmentation and attention enhancements. For majority categories such as “blast” and “normal”, which have larger sample size, the model maintains F1-scores of 0.9465 and 0.9383 respectively, suggesting effective feature learning and generalization even in the presence of higher intra-class variation. However, the model shows relatively lower recall (0.9060) in the “downy_mildew” category, which may be attributed to either subtle inter-class feature differences or the limited number of representative samples. This suggests that some positive instances in this category were misclassified, possibly due to insufficient learning of discriminative cues. Future work may consider strategies such as targeted data augmentation, re-weighted loss functions, or category-specific attention refinement to further improve recall for this class.

Overall, the model achieves F1-scores above 0.90 for all 10 categories, with 6 categories exceeding 0.95, underscoring the effectiveness of the structural modification, including lightweight design, Swish activation, and SE attention.

### 3.2 Comparison of LiSA-MobileNetV2 with traditional CNN models

To further verify the effectiveness of the proposed LiSA-MobileNetV2, we conducted comparative experiments against a range of lightweight and classic convolutional neural network (CNN) models (**Table 4**). The selected baseline models include traditional lightweight architectures such as MobileNetV2, MobileNetV3, ShuffleNet, and GhostNet, as well as deeper and more complex models like InceptionV3 and ResNet18. In addition, two state-of-the-art mobile-friendly architectures, EfficientNet-Lite0 and MobileViT-XXS (extra small variant), were included to benchmark the performance of the proposed model against modern CNN design paradigms.

**Table 4:** Comparison of performance among different CNN models in rice disease classification.

Model	Test Accuracy (%)	FLOPs (M)	Params (K)
MobileNetV2	89.91	639.24	2271.95
MobileNetV3	91.45	121.27	1683.70
ShuffleNet	90.20	259.75	1859.39
GhostNet	94.04	330.54	2601.93
InceptionV3	95.58	5693.41	21823.27
ResNet18	94.14	3634.71	11191.24
EfficientNetLite0	90.87	754.17	3324.46
MobileViT-XXS	95.94	886.91	1087.34
LiSA-MobileNetV2	95.68	331.27	575.06

According to the results in **Table 4**, LiSA-MobileNetV2 achieved the highest test accuracy of 95.68%, while maintaining an ultra-lightweight structure with only 575.06K parameters and 331.27M FLOPs. Compared with the original MobileNetV2, the proposed model improved accuracy by 5.77% while significantly reducing the number of parameters. Although GhostNet and ResNet18 also achieved competitive accuracies (94.04% and 94.14%, respectively), their computational and memory demands were much higher. InceptionV3 yielded a comparable accuracy of 95.58%, but its deployment was hindered by its large size (21.8M parameters and 5693.41M FLOPs). While ShuffleNet, MobileNetV3, and EfficientNet-Lite0 exhibited lower computational overhead, they fell short in classification performance. MobileViT-XXS achieved a slightly higher accuracy (95.94%) than our model but at the cost of significantly increased computational complexity. In summary, the LiSA-MobileNetV2 model demonstrates an excellent trade-off between classification accuracy and computational efficiency, making it particular suitable for deployment in edge and mobile scenarios where resource constraints are critical.

### 3.3 Classification accuracy and computational efficiency from ablation experiments

To systematically evaluate the effectiveness of each structural modification introduced in this study, we conducted a series of ablation experiments focusing on model simplification, the SE attention mechanism, and the Swish activation function (**Table 5**). Each variant model was trained and evaluated five times using different random seeds to ensure statistical robustness. The reported accuracy and precision values represent the mean and standard deviation across these five independent runs.

**Table 5:** Performance comparison of model variants in ablation experiments.

Model	Accuracy (%)	Precision (%)	FLOPs (M)	Params (K)
Li-MobileNetV2	92.19 ± 0.39	92.48 ± 0.38	321.18	570.81
LiA-MobileNetV2	94.50 ± 0.38	94.82 ± 0.38	321.29	576.95
LiS-MobileNetV2	93.85 ± 0.49	94.17 ± 0.47	331.17	570.81
LiSA-MobileNetV2	95.77 ± 0.21	95.84 ± 0.20	331.27	575.06

Accuracy and precision were reported as the mean ± standard deviation across five independent runs with different random seeds.

The results show that the lightweight architecture (Li-MobileNetV2) achieved an average accuracy of 92.19 ± 0.39 and a precision of 92.48 ± 0.38, representing a 2.41% improvement over the original MobileNetV2 baseline (89.91). Introducing the SE attention mechanism (LiA-MobileNetV2) further improved the performance, achieving 94.50 ± 0.38 accuracy and 94.82 ± 0.38 precision, indicating enhanced feature representation and discrimination. When only the Swish activation function was applied (LiS-MobileNetV2), the model reached 93.85± 0.49 accuracy and 94.17 ± 0.47 precision, showing a moderate performance gain. Although the effect is slightly less than that of the SE attention mechanism, it still demonstrate the effectiveness of replacing ReLU6. The combined model (LiSA-MobileNetV2), integrating both SE Swish, yielded the best performance, achieving an average test accuracy of 95.77 ± 0.21 and precision of 95.84 ± 0.20. This confirms the complementary effect of the two enhancements in boosting classification capability. Notably, these improvements incur only a marginal increase in FLOPs and parameters, validating the model’s suitability for deployment in resource-constrained environments.

### 3.4 Generalization to external dataset

To further assess the generalization capability of the proposed model, we conducted cross-dataset evaluation using the Rice Leaf Disease Images dataset from Kaggle, which contains 5,932 images spanning four categories: bacterial blight, blast, brown spot, and tungro. To simulate a low-resource scenario, 50 images per class (200 in total) were randomly selected and incorporated into the original dataset, with 80% added to the training set and 20% to the validation set. The remaining 5,732 images were used as the test set. During fine-tuning, only the final classification layer of the LiSA-MobileNetV2 model was modified to adapt to the four-category classification task, while all other layers were kept frozen. The model was trained for 10 epochs on the selected subset.

**Figure 4:**
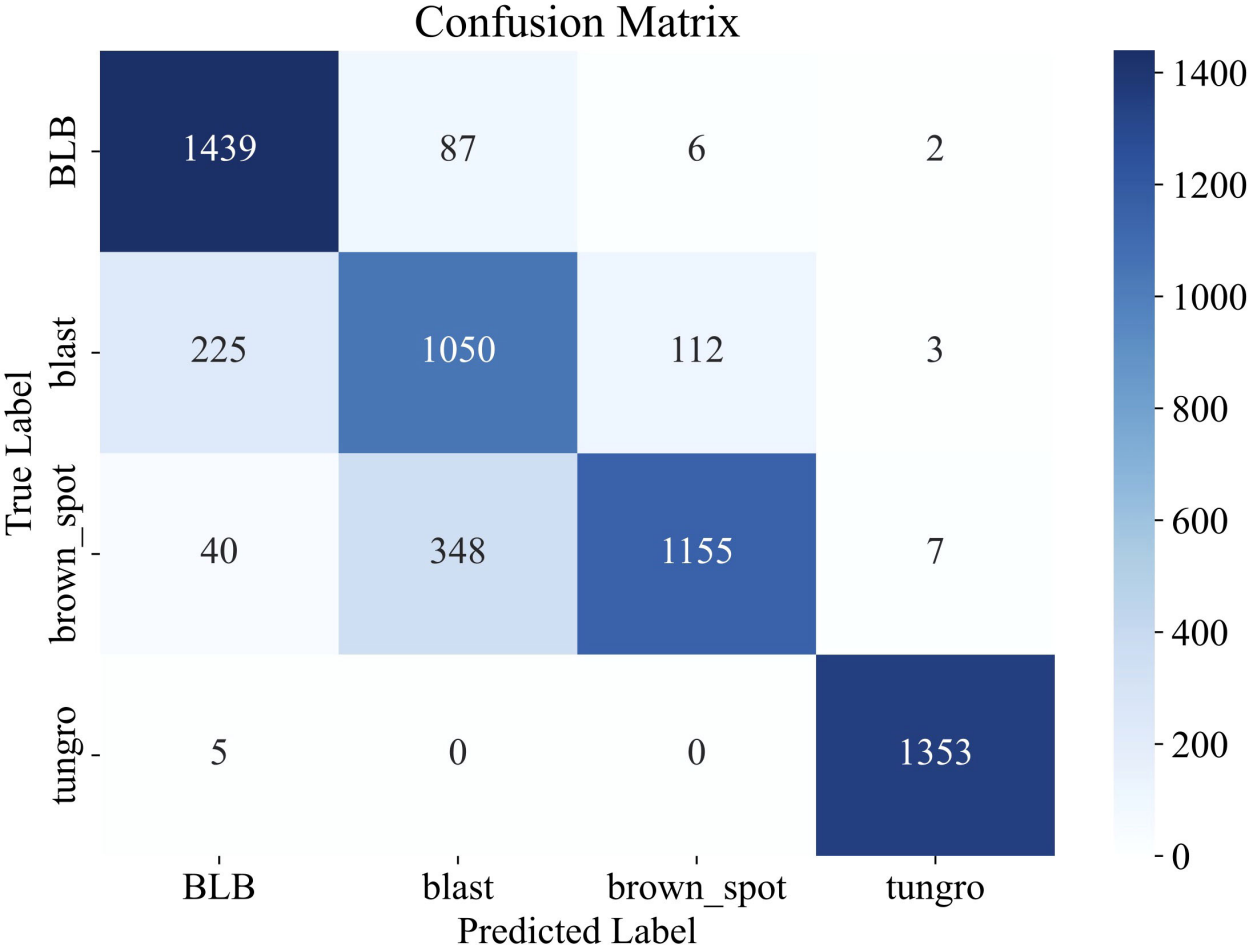
Confusion matrix illustrating the classification performance of LiSA-MobileNetV2 on the external Rice Leaf Disease Images dataset.

Evaluation results on the external dataset demonstrate that our model achieved strong generalization performance, attaining an overall test accuracy of 85.0% (**Figure 4**). Specifically, the model performed exceptionally well on the ‘tungro’ class with over 99% precision and recall and achieved F1-scores of 0.90 and 0.80 on ‘bacterial blight’ and ‘brown spot’, respectively. The macro-average F1-score reached 0.86, highlighting the robustness and transferability of the model under domain shift.

## 4 Discussion

### 4.1 Impact of model architecture optimization on disease recognition

In this study, the network structure of MobileNetV2 was specifically adjusted for the task of rice disease classification by optimizing the settings of the number of channels and the expansion factors within the inverted residual blocks. In Group 1 and 2, relatively small channel numbers (16 and 24) and moderate expansion factors (2 and 3) were used to reduce the computational complexity of the shallow layers. In contrast, deeper blocks such as Group 4 and 5 adopted higher channel counts (64 and 96) and larger expansion factors (6 and 8) to enhance the network’s capacity for learning high-level semantic features.

The results show that the adjusted Li-MobileNetV2 model achieved substantial improvements in multiple metrics compared with the original MobileNetV2. Specifically, classification accuracy increased from 89.91% to 92.32%, a gain of 2.41 percentage points. Meanwhile, the model’s computational cost (FLOPs) was reduced from 639.24M to 321.18M (a 49.78% reduction), and the number of parameters decreased from 2271.95K to 570.81K (a 74.88% reduction), significantly shrinking the model size (**Tables 4** and **5**). These results indicate that Li-MobileNetV2 can maintain or even improve performance while drastically reducing computational and storage requirements.

In terms of depth simplification, we also explored the impact of reducing the number of inverted residual blocks from the original 19 down to 15, 13, and 11. We found that the this reduction significantly affects the classification performance, computational efficiency, and model size (**Table 6**). Among the tested configurations, the 15-block version achieved the highest accuracy (92.51%), but also came with increased FLOPs (371.61M) and parameters (733.34K). Reducing the number of blocks to 13 resulted in a minimal accuracy drop of only 0.19%, while simultaneously decreasing FLOPs by 13.6% and parameters by 22%, demonstrating a better trade-off between accuracy and efficiency. However, further reduction to 11 blocks led to a notable decline in performance (accuracy drops to 89.53%), indicating insufficient feature extraction capability. Therefore, the 13-block configuration offers the most balanced performance, achieving high accuracy with significantly reduced computational demands, making it an ideal choice for practical applications with limited hardware resources.

**Table 6:** Performance of MobileNetV2 model with different numbers of inverted residual blocks.

No. blocks	Accuracy (%)	Flops (M)	Params (K)
15	92.51	371.61	733.34
13	92.32	321.18	570.81
11	89.53	273.35	510.10

### 4.2 Comparison and analysis of convergence performance between Swish and ReLU6

**Figure 5:**
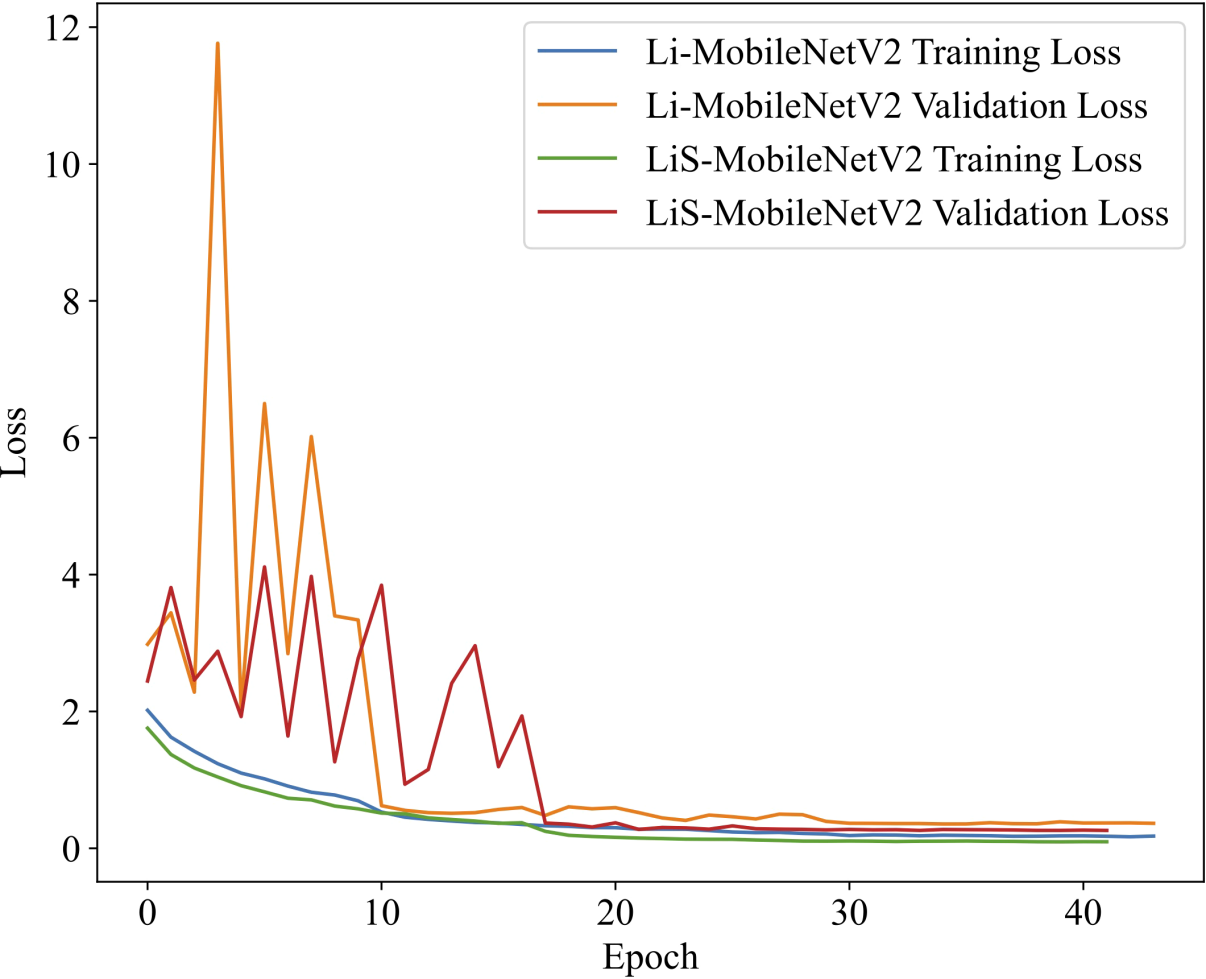
Comparison of loss curves on the training and validation sets using ReLU6 and Swish activation functions. The model with Swish (LiS-MobileNetV2) demonstrates faster convergence and more stable validation performance, indicating improved generalization and training efficiency compared to ReLU6 (Li-MobileNetV2).

The advantages of the Swish activation function over ReLU6 can be validated in terms of training convergence and generalization ability by comparing the training dynamics of LiS-MobileNetV2 (using Swish) and Li-MobileNetV2 (using ReLU6) (**Figure 5**).

The model using the Swish converges noticeably faster during the early training stages and exhibits a more rapid decline in loss compared to the ReLU6-based model. In later stages, the validation loss of the Swish model remains more stable and shows fewer fluctuations, indicating better generalization. Furthermore, the final validation loss of the Swish model is significantly lower than that of ReLU6 model, further demonstrating the enhanced convergence stability and robustness. In summary, the Swish activation function not only improves the final classification accuracy but also exhibits faster and more stable convergence during training, thereby supporting its rationale as a replacement for ReLU6 in lightweight CNN architectures.

### 4.3 Determination of the optimal attention mechanism

To determine the most effective attention mechanism for rice disease classification, we respectively integrated SE (Hu et al., 2018), CBAM (Convolutional Block Attention Module) (Woo et al., 2018), and ECA (Efficient Channel Attention) (Wang et al., 2020) modules into the Li-MobileNetV2 architecture with adjusted inverted residual blocks. The goal was to evaluate their impacts on classification accuracy and precision. The results show that all three mechanisms led to performance improvements to varying degrees (**Table 7**). Among them, the SE attention module delivered the highest performance, achieving a test accuracy of 94.81% and a precision of 94.98%, representing improvements of 2.49% and 2.53% over the baseline Li-MobileNetV2, respectively. CBAM achieved moderate gains (2.11% in accuracy, 2.05% in precision), while ECA offered relatively modest improvements.

**Table 7:** Comparative performance of LiMobileNetV2 with different attention mechanisms.

Model	Accuracy (%)	Precision (%)	Flops (M)	Params (K)
Li-MobileNetV2	92.32	92.45	321.18	57.08
LiA-MobileNetV2	94.81	94.98	321.29	57.70
Li-CBAM-MobileNetV2	94.43	94.50	321.64	57.51
Li-ECA-MobileNetV2	92.99	93.18	321.28	57.08

We hypothesize that the superior performance of SE arises from the nature of rice disease features, which typically manifest as channel-sensitive variations (e.g., color intensity, lesion density, and texture patterns) rather than complex spatial arrangements. Unlike CBAM, which combines both spatial and channel attention, SE focuses purely on global channel dependencies, enhancing feature recalibration without introducing additional complexity of spatial modeling. Compared to ECA, SE also captures broader contextual relationships across channels, improving discriminative capacity while maintaining low computational cost. These results suggest that SE attention is particularly well-suited for rice disease image recognition tasks, where visual symptoms are more dependent on channel-level cues than spatial localization patterns.

### 4.4 Analysis of class-wise classification accuracy using confusion matrix

**Figure 6:**
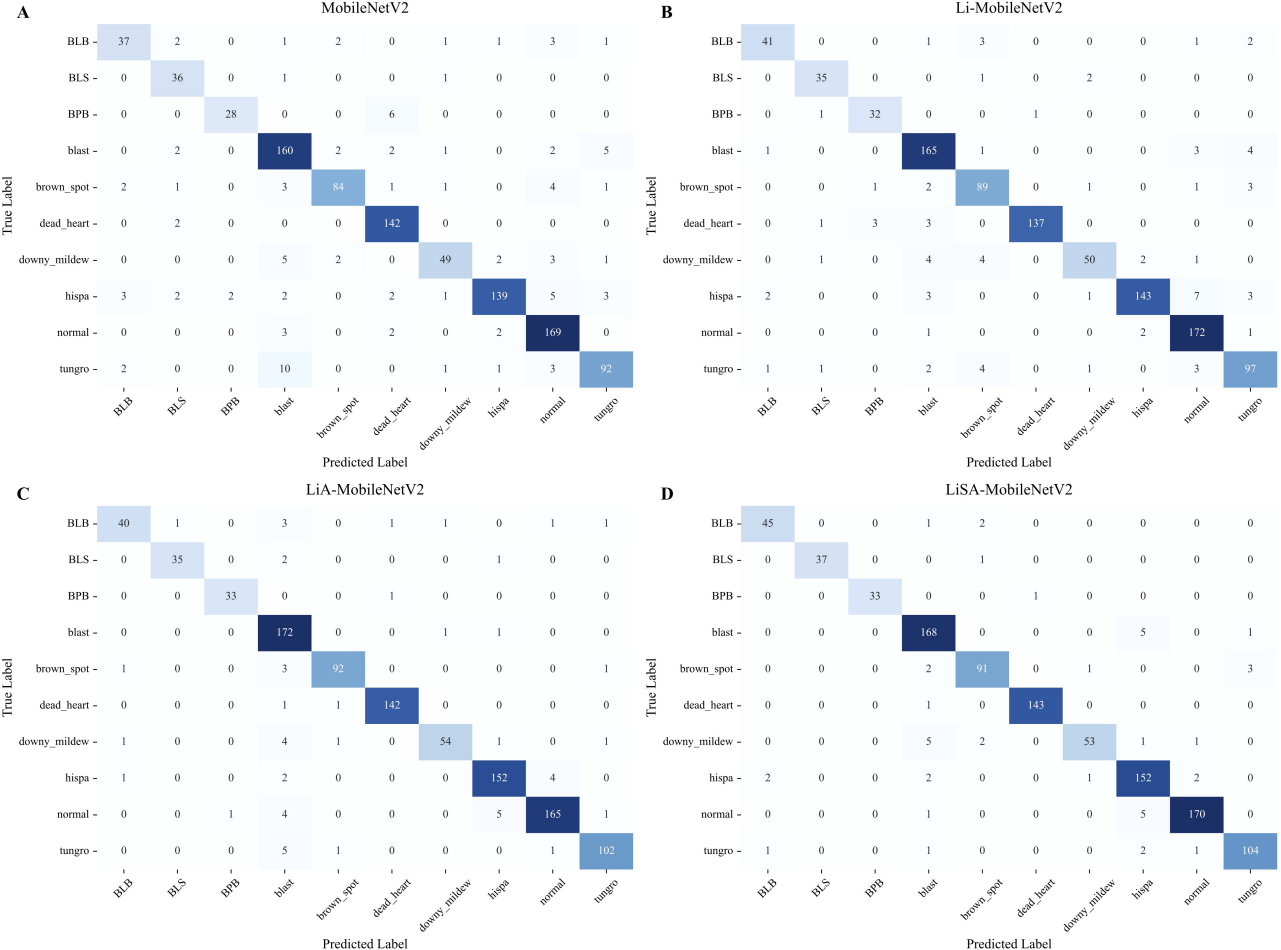
Comparison of confusion matrices for rice disease classification using the original MobileNetV2 **(A)** and its three improved variants: Li-MobileNetV2 **(B)**, LiA-MobileNetV2 **(C)**, and LiSA-MobileNetV2 **(D)**.

The performance improvements of the proposed variants of MobileNetV2 (**Table 2**) were analyzed in a class-wise manner using confusion matrices (**Figure 6**).

The result indicates that LiSA-MobilNetV2, incorporating both the SE attention mechanism and Swish activation, consistently achieved the highest classification accuracy across most disease classes. In particular, the model attained 100% accuracy for BLS and BPB, with a lower overall misclassification rate than its counterparts, confirming the effectiveness of the proposed architectural enhancements (**Figure 6D**). In contrast, the original MobileNetV2 shows poor recognition rate for several diseases. For instance, 10 ‘tungro’ samples are misclassified as ‘blast’, suggesting insufficient feature extraction for visually similar symptoms (**Figure 6A**). By modifying the inverted residual blocks, the improved Li-MobileNetV2 enhanced classification accuracy for ‘blast’, ‘brown spot’, and ‘tungro’ (**Figure 6B**). Introducing the SE attention module further improved recognition of ‘blast’, ‘hispa’, and ‘tungro’, indicating that channel-wise feature recalibration helps the model focus on disease-relevant patterns (**Figure 6C**). Finally, integrating the Swish activation function provides additional performance gains. Although there was a marginal decrease in accuracy for ‘blast’, ‘brown spot’, and ‘downy midew’, the recognition of other classes improved, demonstrating that Swish generally enhances the model’s representational capacity (**Figure 6D**).

Among all categories, ‘downy mildew’ remains the most challenging to classify, exhibiting the lowest precision and recall (**Figure 6**). Several factors may contribute to this: 1) visual similarity to early-stage symptoms of ‘blast’, especially under natural lighting, can cause misclassification due to subtle chromatic differences; 2) high intra-class variability from pale lesions to necrotic patches complicates consistent feature learning; 3) despite oversampling, the relatively small sample size limit the diversity of training examples and reduces generalization. These findings suggest that further enhancement could involve more targeted data augmentation, incorporation of spatial attention modules, or multi-scale feature fusion strategies to improve discrimination for visually ambiguous classes like Downy mildew.

### 4.5 Interpretation of improved models via Grad-CAM visualization

**Figure 7:**
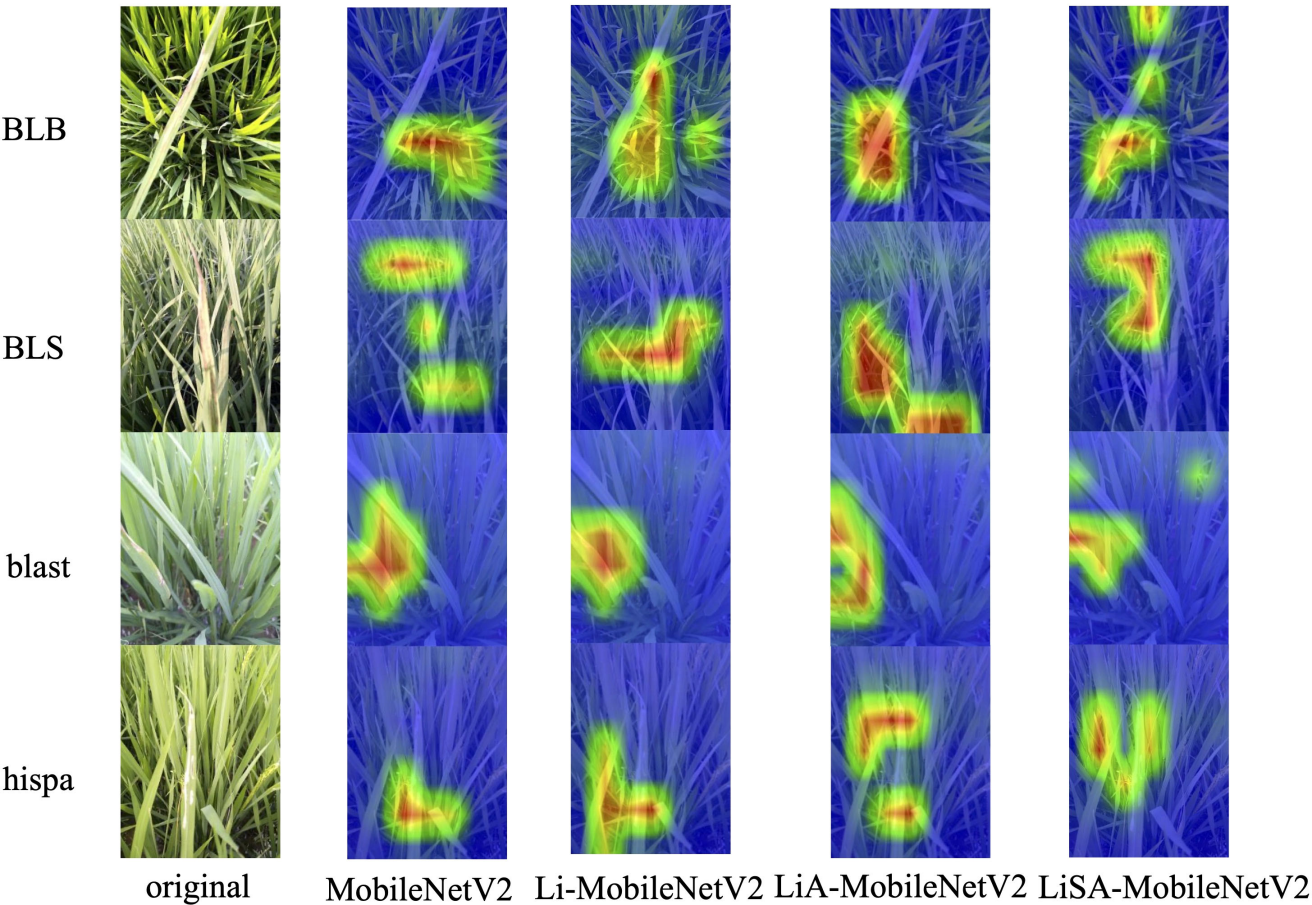
Grad-CAM visualizations for the original MobileNetV2 and its improved variants across four representative rice disease categories. The improved models, especially LiSA-MobileNetV2, demonstrate stronger and more accurate focus on disease-affected regions, reflecting enhanced feature extraction and classification interpretability.

To enhance the interpretability of the proposed models and verify their effectiveness in rice disease classification, Gradient-weighted Class Activation Mapping (Grad-CAM) was employed to generate visual explanations (Selvaraju et al., 2017). Grad-CAM highlights the most influential regions in the input image by utilizing the gradients of the predicted class with respect to the activations in the last convolutional layer, thereby producing heatmaps indicating the model’s focus during inference. This analysis covers four categories with varying classification performance: ‘blast’ and ‘hispa’, which have relatively large test sample sizes (174 and 159 samples in the test set, respectively); ‘BLS’, which exhibits high classification accuracy across all models (≥35 correct predictions out of 38); ‘BLB’, a relatively challenging class where the original MobileNetV2 model achieved only 77.08% accuracy. Grad-CAM results for representative images are shown in **Figure 7**, where red indicates regions of high attention and blue indicates low attention.

From the visualizations, we found that for the BLB disease, the original MobileNetV2 fails to accurately localize the disease lesions, with attention scattered across irrelevant background areas. Li-MobileNetV2 shows slight improvement, but still suffers from background distraction. In contrast, LiA-MobileNetV2 and LiSA-MobileNetV2, both equipped with SE attention, successfully focus on the diseased areas, with LiSA-MobileNetV2 providing the most precise localization. For the BLS disease, all models exhibit clear and consistent focus on disease-relevant areas, corresponding with their high classification accuracy, indicating strong discriminative capability regardless of model variant. Regarding the ‘blast’ and ‘hispa’, the baseline and Li-MobileNetV2 models display narrow and incomplete attention coverage, whereas the attention-augmented models (LiA and LiSA) demonstrate broader and more comprehensive focus across the diseased regions. Notably, LiSA-MobileNetV2 delivers the most coherent and interpretable attention maps.

These results confirm that the combination of the Swish activation function and the SE attention mechanism significantly improves not only the model’s classification performance but also its ability to concentrate on semantically meaningful regions in the image.

## 5 Conclusions

In this study, we proposed an enhanced scheme for improving MobileNetV2’s performance in rice disease classification. By simplifying the inverted residual structure, introducing the Swish activation function, and incorporating the SE attention mechanism, we effectively optimized the model’s feature extraction capability. These improvements not only enhanced classification but also further reduced the model’s computational load, making it more suitable for deployment in resource-constrained environments.

Experimental results demonstrated that the proposed LiSA-MobileNetV2 model achieved a classification accuracy of 95.68% on the test set, an improvement of 5.77% over the original MobileNetV2, while significantly reducing the number of parameters and computational overhead. Comparative experiments with different attention mechanisms (SE, ECA, and CBAM) further confirmed that the SE module offers the most effective performance gains in this task.

Looking forward, we plant to address the misalignment occasionally observed between attention regions and actual lesion areas, particularly in the cases of ‘blast’ and ‘hispa’ diseases. This issue may stem from limitations in feature representation caused by the lightweight design, lack of pixel-level annotations, or inter-disease visual similarities. Future word will explore the incorporation of fine-grained supervision, attention refinement modules, or weakly supervised learning techniques to improve interpretability and localization accuracy. Additionally, the optimized model will be deployed on mobile and edge computing platforms to evaluate its performance in real-world agricultural settings, with further efforts to optimize inference speed and system responsiveness.

## Data Availability

Publicly available datasets were analyzed in this study. This data can be found here: www.kaggle.com/competitions/paddy-disease-classification.
